# Long-Term Atmospheric Corrosion Behavior of Epoxy Prime Coated Aluminum Alloy 7075-T6 in Coastal Environment

**DOI:** 10.3390/ma11060965

**Published:** 2018-06-07

**Authors:** Sheng Zhang, Yuting He, Teng Zhang, Guirong Wang, Xu Du

**Affiliations:** 1 Aeronautics Engineering College, Air Force Engineering University, Xi’an 710038, China; zs_xinzhou@126.com (S.Z.); zt_gm@126.com (T.Z.); duxu_1990@126.com (X.D.); 2 College of Materials Science and Chemical Engineering, Harbin Engineering University, Harbin 150001, China; wgr5150@126.com

**Keywords:** epoxy prime, aluminum, EIS, EPMA, atmospheric corrosion, exfoliation corrosion

## Abstract

The atmospheric corrosion of epoxy prime coated aluminum alloy 7075-T6 exposed for 7, 12 and 20 years was investigated. The remaining thicknesses of epoxy prime coatings for macroscopically intact coating areas followed a normal distribution and decreased linearly. EIS results demonstrated that the corrosion resistance of the coating decreased with exposure time. After 20 years of exposure, the epoxy coating had lost its protection as cracks existed within the coating and exfoliation corrosion had occurred on the substrate. The substrate was sensitive to exfoliation corrosion through metallographic and TEM analysis. The corrosion products were mainly hydroxides of aluminum. The morphology and chemical compositions of the coating bubbling area and propagation characterizations of exfoliation corrosion were analyzed by SEM, EPMA and EDS. Cracks between the lumps of corrosion products provided the channels for the transmission of corrosion mediums. Furthermore, the mechanical model was proposed to analyze the propagation characterization of exfoliation corrosion.

## 1. Introduction

Because of its high strength and low density, the aluminum alloy 7075-T6 is widely used in aircraft structures; however, the service of aircraft structures in the outdoor atmosphere exposes them to the influence of atmospheric corrosion [1]. To improve the corrosion resistance and safety of aircraft structures, coating systems have been extensively used to protect aluminum alloys against corrosion [2,3].

Generally, aircrafts utilize a three-layer coating system including a conversion coating, primer and topcoat [4]. Currently, the internal structures of military aircrafts mostly utilize the two-layer coating system composed of an anodic oxide film and an epoxy primer. The advantage of epoxy primer is that when water seeps into the coating, the active pigments rapidly hydrolyze, forming ions that passivate the metal surface, effectively preventing metal corrosion [5,6]. On the other hand, although these coating systems initially blocked the diffusion of ions effectively, with the increase of service time, water and aggressive ions gradually penetrated to the metal/coating interface, resulting in substrate corrosion and coating failure [7].

The corrosion of coated metals, organic coatings, and aluminum alloys has been extensively investigated through accelerated tests or electrochemical methods [8,9,10,11,12,13,14]. These laboratory tests are particularly useful for determining the influence of specific pollutants and ions [15], but for evaluating the service performance of structures or materials, field testing in the actual environment is more reliable. Smith et al. reported that the corrosion of a bare 7075-T6 aluminum alloy sheet exposed for 4 years was mild in marine and inland atmospheres [16]. Sun et al. carried out atmospheric corrosion tests on the alclad 7075 aluminum alloy in four different environments in China for 20 years and investigated the mechanical properties and depth of pitting [17]. Katayama et al. investigated the corrosion properties of the carbon steel plates with Zn, Al and Zn-Al thermally sprayed coatings exposed to a coastal area for about 33 years using electrochemical impedance measurements [18]. Different kinds of epoxy coating systems were exposed to the corrosive atmosphere of petrochemical industry for 2 years to evaluate their protective effects and determine the corrosive grade of the corrosive atmosphere [19]. Bano et al. reported the performance of epoxy-polyamide primer and coal tar epoxy topcoat system applied on mild steel which subjected to various natural exposures including marine, industrial and urban test sites of Karachi city for 21 months, while an accelerated test was also conducted for comparison [20]. In order to investigate the corrosion failure characteristic of airplanes served in the tropical coastal environment of Fiji for 1600 flight hours, epoxy prime coated aluminum alloy taken from the airplane was studied by examining the macro and micro images, and analyzing the composition of corrosion products [21]. The results of these atmospheric exposure tests are exceedingly valuable, and have provided the academic and industrial communities with important data; however, there are very few reports about the corrosion behavior of epoxy primer coated aluminum alloys exposed to a coastal environment for a long period of time.

This paper presents the surface appearance and corrosion products of epoxy primer coated aluminum alloy 7075-T6 exposed for 7, 12 and 20 years in the Wanning test site in China. We also estimated the atmospheric corrosion properties of epoxy coatings using a statistical method and electrochemical impedance measurements. The exfoliation corrosion resistance of the aluminum alloy substrate was analyzed by metallographic and TEM analysis. Finally, we analyzed the morphology and chemical compositions of coating bubbling area and propagation characterizations of exfoliation corrosion by SEM, EPMA and EDS.

## 2. Experimental

### 2.1. Specimens

**Figure 1:**
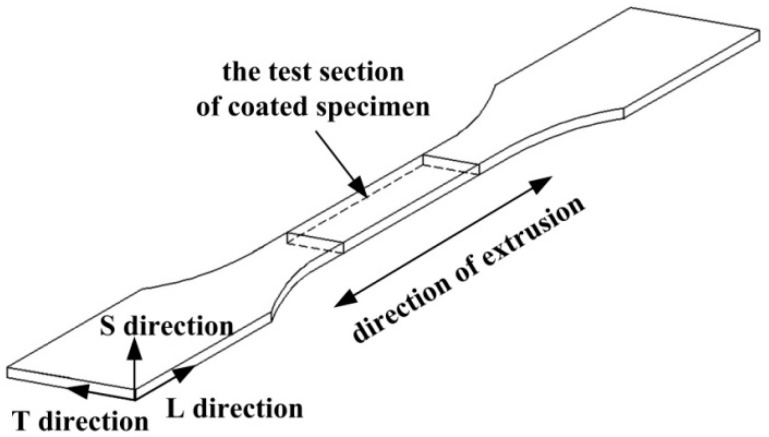
Schematic drawing of the specimen’s test section.

Figure 1 shows the geometry of the specimens exposed to the coastal environment. The intermediate section of the specimen was the corrosion test section with a 220 mm length, 400 mm width, and 4 mm thickness. Extruded 7075-T6 aluminum alloy was selected as the substrate and its chemical composition is given in Table 1. The three dimensions of a microstructure are generally defined as longitudinal (L), long transverse (T), and short transverse (S) and the L direction is identified as the direction of extrusion. The substrates were ultrasonically degreased in acetone, anodized in sulfuric acid, and sealed with hot water. The ultimate thickness of the anodic oxide film was 8 μm. Finally, the zinc chromate pigmented epoxy primer was sprayed within 24 h and the surface of the specimen to be sprayed was perpendicular to the spray gun during spraying. Then the coated specimens were left to dry in a ventilated environment at 40 °C. The average thickness of the coating was controlled at 50 μm. The zinc chromate is a kind of typical anti-corrosion pigments in the coating. Although the epoxy zinc chromate primer had been banned, the epoxy zinc chromate primer was widely applied to protecting the internal aluminum alloy structures of military aircrafts against corrosion before 1997. The research of this primer is of great significance for evaluating the structural safety of aging aircraft serving in coastal environments for a long period of time.

### 2.2. Field Exposure Test

**Figure 2:**
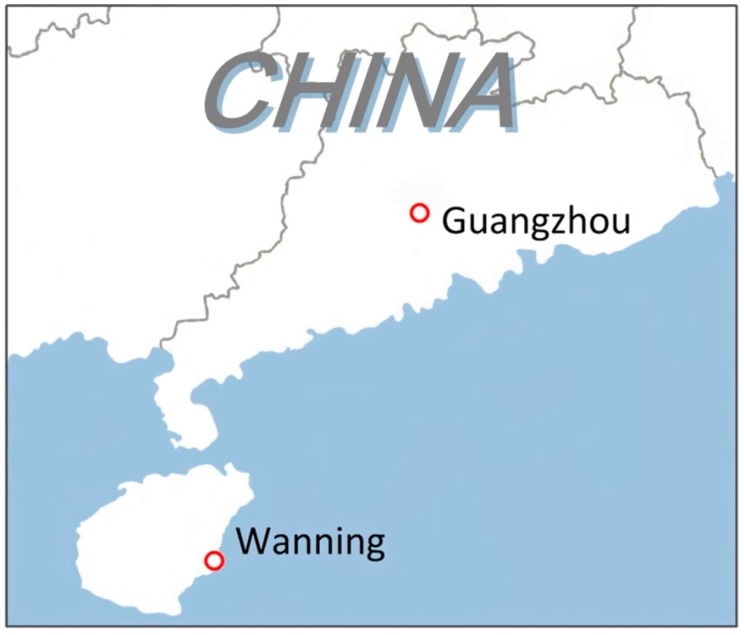
Location of the exposure test site.

The exposure tests were performed in the Wanning test site in China. Figure 2 shows the location of the Wanning test site. Table 2 provides the average environmental data measured in 1997 at Wanning and the annual environmental data can be found free in this website named “China Gateway to Corrosion and Protection” whose URL is http://data.ecorr.org/edata/01/0103/010301/01030101/index.html. All the specimens were placed on the test racks facing south at an angle of 45° in a ventilated room to simulate the environment in which the internal structures of an aircraft were located. The analyses were then carried out on the upper surface, facing the sky, since it had endured more pronounced aging and corrosion. Five specimens were withdrawn from the test sites after 7, 12 and 20 years of exposure for each time period and then kept in a desiccator.

### 2.3. Surface and Corrosion Products Analyses

The upper surface appearance of the test section (defined in Figure 1) of the specimens with different years exposure was analyzed using a PXS-5T stereomicroscope with an image sensor. After microscopic observation, the upper surface corrosion products in substrate corrosion area of a specimen exposed for 20 years were identified by X-ray diffraction using an X’Pert PRO.

### 2.4. Electrochemical Impedance Measurement

Electrochemical impedance spectroscopy (EIS) measurements were performed using a PARSTAT 2273 electrochemical workstation (AMETEK, Princeton, NJ, USA). The electrochemical impedance test samples with a coating area of 1 cm^2^ were taken from the macroscopically intact coating area on the test section of the specimens. A three-electrode cell arrangement was used in the experiments, with the coated sample electrode as working electrode (WE), a platinum wire as counter electrode (CE) and a saturated calomel electrode (SCE) as reference electrode (RE). The electrolyte employed in this measurement was a 3.5% NaCl solution. The impedance data was taken after measuring the open circuit potential for several tens of seconds. The impedances were characterized over frequencies ranging from 100 kHz to 10 mHz, using a 20 mV perturbation signal. Equivalent circuit modeling was performed using ZSimpWin software (EChem Software, Ann Arbor, MI, USA).

### 2.5. Sectional Analyses

#### 2.5.1. Measurement of Thickness of Coating

**Figure 3:**
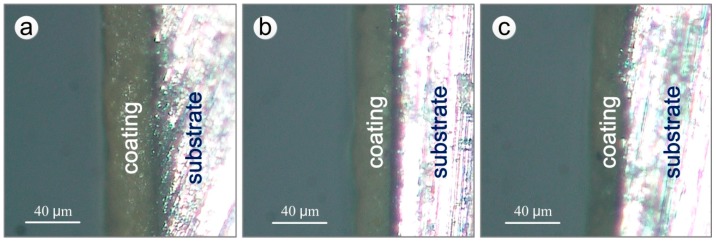
Cross-sections of specimens exposed for different years (one side of the upper surface coatings): (**a**) 7 years; (**b**) 12 years; (**c**) 20 years.

The remaining thickness of the epoxy coating in the macroscopically intact coating area on the test section of the specimens after different exposure times was estimated using a statistical method. Slices of coating cross-section from a macroscopically intact area within the test section were ground with a 1000 grit SiC paper. The remaining thickness of the upper surface coating was measured by RH-8800 digital video microscope (HIROX, Tokyo, Japan) and TSview-7 software. Figure 3 shows the cross-sections of one side of the upper surface coating of specimens exposed for different years. For every corrosion period, 6 sections were measured, each of which was divided in 10 evenly spaced regions to measure the remaining thickness of the coating.

#### 2.5.2. Analysis of Exfoliation Corrosion Resistance of Aluminum Alloy Substrate

The S-L section of the extruded 7075 aluminum alloy substrate exposed for 20 years was ground with a 1000 grit SiC paper, polished with 2.5 μm diamond paste, and chemically etched with Keller’s reagent (1.0 mL HF + 2.5 mL HNO_3_ + 1.5 mL HCl + 95 mL H_2_O) for 15 s. The microstructure of the S-L section was then observed by metalloscopy.

A coating test sample with a cross-section thickness of 0.5 mm from a specimen that had been exposed for 20 years was ground with a 2000 grit SiC paper until a thickness of 80 μm was reached (measured with micrometer caliper) was obtained. Thinning and perforation were carried out on 695 PIPS COOL ion beam thinner (Gatan, Pleasanton, CA, USA), thus completing the preparation of TEM samples. The grain boundary structure of the aluminum alloy was observed on a FEI Talos F200X high resolution transmission electron microscope (FEI, Hillsboro, OR, USA).

#### 2.5.3. Corrosion Failure Analysis of Aluminum Alloy Coating System

The S-L sections of the coating bubbling area and of the junction area between the corroded and un-corroded substrate of a specimen exposed for 20 years were ground with a 1000 grit SiC paper and polished with 2.5 μm diamond paste. The sectional morphology and chemical compositions of the corrosion products were analyzed through SEM and EDS. In addition, the elemental distribution of the coating bubbling section was obtained by EPMA.

## 3. Results and Discussion

### 3.1. Surface Appearance and Characterization

**Figure 4:**
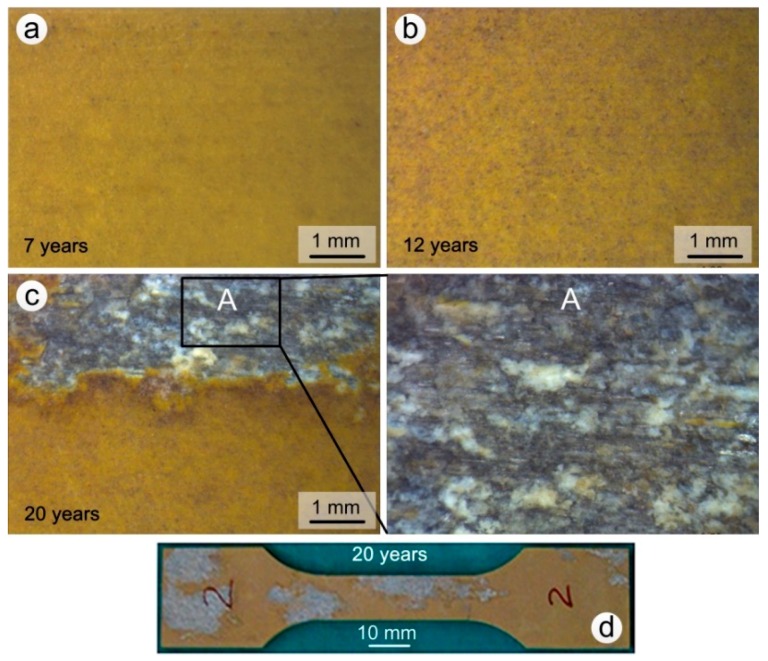
Surface appearance of coated test specimens exposed to the Wanning test site: (**a**) 7 years; (**b**) 12 years; (**c**) 20 years; (**d**) 20 years (whole upper surface).

The surface appearance of coated specimens exposed for 7, 12 and 20 years in the Wanning test site are shown in Figure 4. The degree of aging increased with the exposure time and the color of the coating changed, gradually becoming darker. After 7 years a small amount of salts and impurities were deposited on the surface of the coating (Figure 4a); the deposit present on samples that had been exposed for 12 and 20 years also contained impurities such as sand and dust (Figure 4b,c). The concentration of impurities was higher on samples exposed to the atmosphere for 20 years. The surface of the epoxy coating remained macroscopically intact when the exposure times was lower than or equal to 12 year, and no localized corrosion occurred. On the other hand, after 20 years the epoxy coating had partially lost its protection ability, and both the coating and the anodic oxide film had been completely destroyed in the exfoliation corrosion regions (Figure 4d). Meanwhile, the extruded 7075 aluminum alloy substrate displayed layered peeling and the formation of white corrosion products in box A, which are typical of exfoliation corrosion (Figure 4c). The percentage of corrosion regions in corrosion test section of the most severely corroded specimen is up to 70% after 20 years exposure. At the same time, the average percentage of five specimens is about 30%, which is similar to that of specimen 2# (Figure 4d).

**Figure 5:**
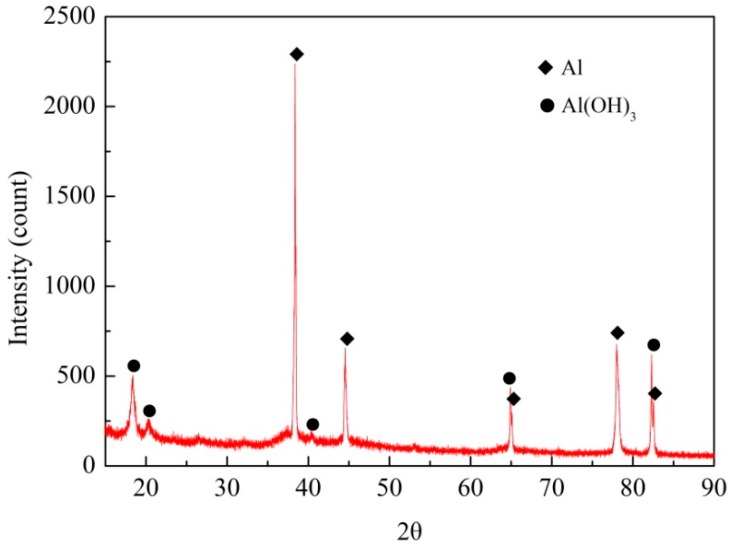
XRD spectrum of exfoliation corrosion products.

The XRD pattern of the white corrosion products in substrate corrosion area of a specimen exposed for 20 years is shown in Figure 5. The results show that the corrosion products were comprised of Al(OH)_3_. The volume of this component is larger than that of the parent material, thus forcing the sheets apart. In addition, the more elongated the grains are, the greater the generated wedging force [1,22,23,24].

### 3.2. Statistical Analysis of the Remaining Thickness of Coating

From Figure 3, it can be observed that the thicknesses of the epoxy coatings decreased noticeably with exposure time, and the outer surface of the coating was smooth, without visible damage. The remaining thicknesses were described through the Normal, Log-normal, Gumbel, Logistic and Weibull distributions. The corresponding distribution functions are shown below:

(1) Normal distribution:
  (1) $$F(x)=\int_{-\infty }^{x}\frac{1}{\sqrt{2\pi }\cdot \sigma }\cdot e^{-\frac{{\left(x-\mu \right)}^{2}}{2\sigma^{2}}}dx$$
(2) Log-normal distribution:
  (2) $$F(x)=\int_{-\infty }^{\mathrm{lg}x}\frac{1}{\sqrt{2\pi }\cdot \sigma }\cdot e^{-\frac{{\left(\mathrm{lg}x-\mu \right)}^{2}}{2\sigma^{2}}}dx$$
(3) Gumbel distribution:
  (3) $$F(x)=\exp[-\exp(-\frac{x-\mu }{\sigma })]$$
(4) Logistic distribution:
  (4) $$F(x)=\frac{\exp[(x-\mu )/\sigma ]}{1+\exp[(x-\mu )/\sigma ]}$$
(5) Weibull distribution:
  (5) $$F(x)=1-\exp[-{\left(\frac{x}{\sigma }\right)}^{\beta }]$$

where *x* is the remaining thickness variable; *μ* is the location parameter which determines the range of abscissa value of the distribution function and indicates the location of the centralized distribution of measured data; *σ* is the scale parameter which determines the scale of the distribution function and indicates the discreteness of measured data; *β* is the shape parameter which determines the shape of the distribution function and directly changes the property of the distribution function. Changes of *σ* only compress or expand the distribution function without changing its basic shape. After the logarithmic linearization of these five distributions, the different parameter values were obtained by the least square fitting [25] and the fitting degrees were compared through the Pearson correlation coefficient (*r*). Table 3 shows the fitting parameters and correlation coefficients corresponding to the different distributions.

According to the relationship between the correlation coefficient and the *t*-distribution, the critical correlation coefficient (*r_c_*) that satisfies distribution assumption can be calculated by [26]:

(6) $$r_{c}=\frac{t_{\alpha }(n-2)}{\sqrt{\left(n-2)+t_{\alpha }^{2}(n-2\right)}}$$

**Figure 6:**
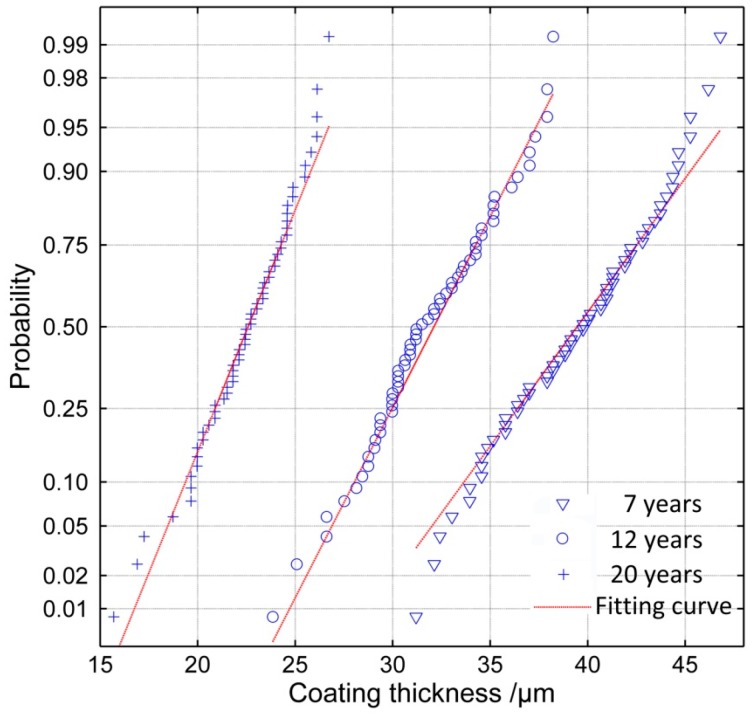
Result of the probability paper test of normal distribution (20 years’ data from coating-still-existing areas).

When the sample size (*n*) is 60 and the significance level *α* is 0.01, the *r_c_* is 0.330. It can be seen in Table 3 that all the distributions have a good correlation with the remaining thickness; however, the data is fitted most effectively by the normal distribution function, while the Gumbel distribution describes the data least effectively. The probability paper test of normal distribution of the remaining thickness is shown in Figure 6 and these data for 20 years are from areas where coating still existed (about 70% of the surface). According to the results, it can be concluded that the remaining thickness of the epoxy coatings exposed over 7 years satisfies a normal distribution.

The remaining thickness to be considered for the practical application of the coating was then determined by defining a reliability (*p_s_*) and confidence level (*γ*). For fixed values of *p_s_* and *γ*, the remaining thickness (*t*) can be calculated by:

(7) $$t=\bar{t}+ks=\frac{1}{n}\sum t_{i}+k{\left[\frac{\sum t_{i}^{2}-n{\left(\frac{1}{n}\sum t_{i}\right)}^{2}}{n-1}\right]}^{0.5}$$

In Equation (7), $\bar{t}$ is the thickness average, *s* is the thickness standard deviation, *n* is the sample size, and *k* is a one-side tolerance coefficient that can be calculated using the methods reported previously [26].

**Figure 7:**
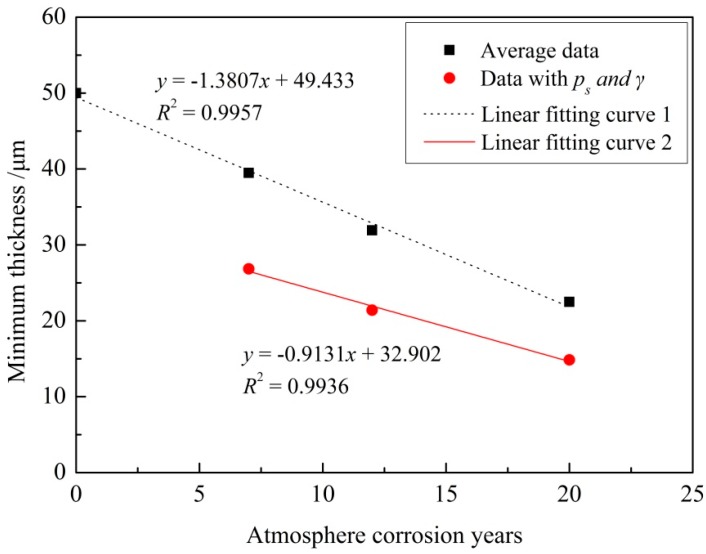
Relationship between atmospheric corrosion exposure years and remaining thickness (20 years’ data from coating-still-existing areas).

In this paper, *n* is 60, and *k* is −3.237 corresponding to the general reliability of 99.9% and confidence level of 95%. The estimated thickness of the epoxy coating exposed for 7 years is 39.5 μm, as calculated by Equation (7). This means there is a 95% probability that the coating thickness will be over 39.5 μm in more than 99.9% of the regions after 7 years of exposure [27]. Figure 7 shows the average remaining thickness and the remaining thickness with 99.9% reliability and 95% confidence level of epoxy coatings in the macroscopically intact coating area on the test section of the specimens exposed for different times. The average data in Figure 7 means the arithmetic mean value of the measured thickness data and these data for 20 years are from areas where coating still existed.

It can be seen that the remaining thicknesses of epoxy coatings decrease linearly as the exposure time increases (Figure 7). As those thickness data for 20 years are from areas where coating still existed, this linear degradation relationship is only valid for macroscopically intact coating areas. This linear degradation phenomenon may be mainly due to the fact that yearly variations of the atmospheric environment are negligible, although there are seasonal and daily variations. Thus the rate of aging and corrosion and of thickness decrease of the coating was constant.

### 3.3. Electrochemical Characterization of Coated Samples with Different Exposure Years

**Figure 8:**
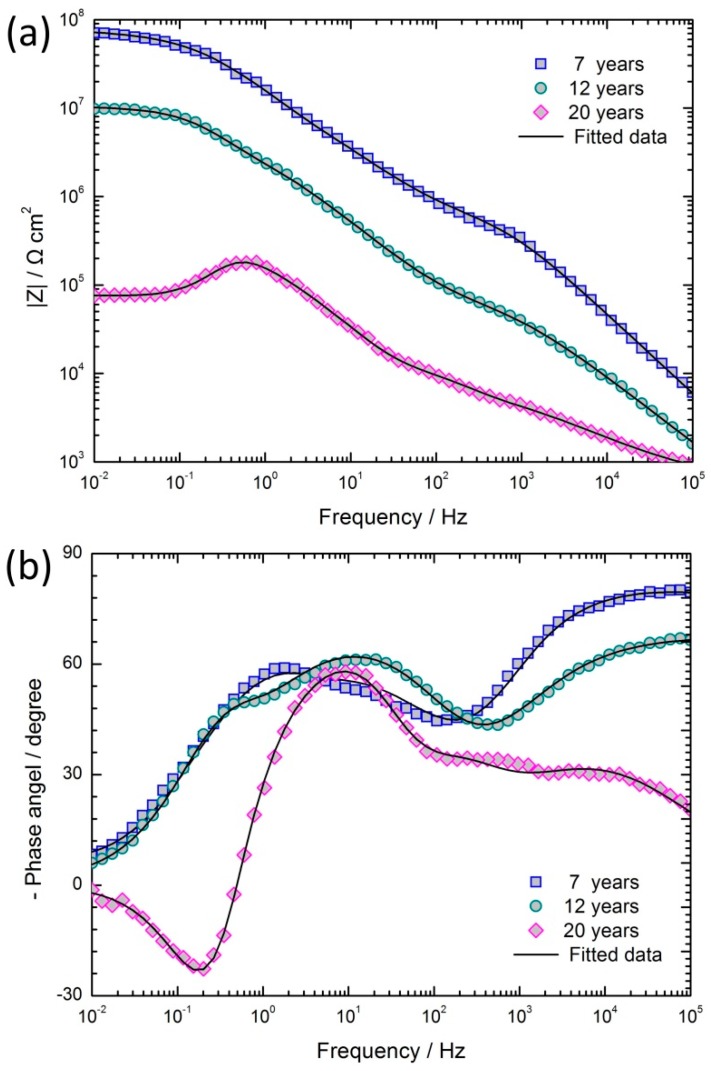
EIS results and its fitted data of coated specimens exposed for 7, 12, and 20 years: (**a**) Bode log |Z| versus log (f/Hz) plots; (**b**) Bode phase angle versus log (f/Hz) plots.

The experimental Bode plots of coated samples after different exposure periods are shown in Figure 8, where the experimental data (scatter plot) can be seen to be accurately described by the fitted data (curves). The Bode log |Z| and the Bode phase angle plots can better explain the frequency specific impedance behavior. From the Bode log |Z| plots (Figure 8a), it may be observed that the oblique lines of the impedance curves at high frequency, corresponding to the coating capacitance, shift towards lower frequencies with the exposure time. This result indicates that the capacitance of the coating increased and its corrosion resistance decreased with the increase in exposure time. In addition, as the coastal exposure time increased, the values of the impedance at 10 mHz, |Z|_f=0.01_, decreased significantly from 70.8 MΩ cm^2^ to 0.075 MΩ cm^2^. After 12 years of exposure, the impedance of the coating still had a higher value, and the epoxy zinc chromate coating had a protective effect on substrate. On the contrary, after 20 years of exposure, the phase angle was less than zero at the low frequency region (Figure 8b). This phenomenon shows the inductive characteristics, and indicates that the coating has lost its capability of protecting the substrate.

The Bode log |Z| plots in Figure 8a are all described by lines with slopes between −0.5 and −0.2 in the middle frequency section, where a linear platform should appear. This characteristic of the coated specimen usually occurs in the middle stage of immersion and is called the Warburg impedance characteristic [28]. The production of Warburg impedance is due to the resistance of additive particles in organic coatings: the electrolyte seeps into the interior from gaps between particles along the winding routes, rather than along the direction of the concentration gradient. This phenomenon is also known as ‘tangent direction diffusion’ [29]. According to the characteristics of the epoxy zinc chromate coating, the phenomenon may be due to the addition of zinc chrome pigments into the coating. When the electrolyte seeps into the coating, zinc chromates will hydrolyze and generate K_2_CrO_4_, ZnCrO_4_, Zn(OH)_2_ and Zn_2_(OH)_2_CrO_4_. A large number of zinc chromate pigments and their hydrolysis products contribute to increase the transport resistance, making it more difficult for the electrolyte to seep into the coating and slowing down the transmission of reaction particles to the corrosion interface, thus causing the diffusion impedance [13].

The Bode phase angle plots of coated samples exposed for 12 and 20 years showed the existence of three time constants. When the electrolyte reached the anodic oxide film surface through the coating, the chlorine ions accelerated the dissolution of the anodic oxide film, and the corrosion of the aluminum alloy substrate occurred at the site of the destruction of anodic oxide film [30]. Therefore, the three time constants corresponded to the time constant of the impedance of coating, the time constant of the dissolution of the anodic oxide film, and the time constant of the corrosion of the aluminum alloy substrate.

**Figure 9:**
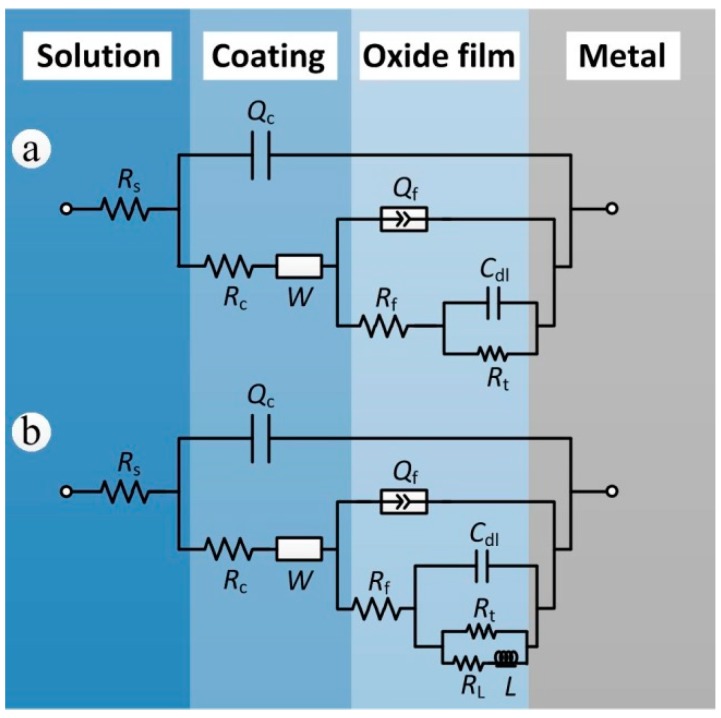
Electrochemical equivalent circuits for fitting the impedance data: (**a**) 7 and 12 years; (**b**) 20 years.

Although the compositions of the coated samples after exposure for 7, 12, 20 of years were basically the same, since the EIS results of samples exposed for 20 years showed inductance characteristics it became necessary to add inductance and inductive resistance in the equivalent electrical circuit selection of samples exposed for 20 years [31]. The equivalent circuits that were found to most closely reproduce the impedance diagrams are shown in Figure 9. The curves were fitted using the ZSimpWin software. The elements of the two chosen equivalent electrical circuits are as follows: *R*_s_ is the solution resistance, *R*_c_ and *Q*_c_ are the coating resistance and coating capacitance, respectively, and *W* represents the Warburg diffusion impedance of the coating. The parameter groups *Q*_f_ and *R*_f_ represent the interface double layer capacitance and the charge transfer resistance of anodic oxide film corrosion reaction, respectively. The parameter groups *C*_dl_ and *R*_t_ represent the charge-transfer reaction at the electrolyte/substrate aluminum alloy. *L* and *R*_L_ respectively represent the inductance and inductive resistance produced by substrate corrosion. The inductive loop is usually obtained by the corrosion products deposited or adsorbed on the sample surface [32,33].

In this work, the frequency response characteristics of the coating and anodic oxide film were not consistent with the pure capacitance; therefore, the capacitance elements were fitted by constant phase elements *Q* (CPE) to compensate for non-ideal capacitance [29]. The impedance of *Q* is given by the following equation [18,34].

(8) $$Z_{Q}=Y_{0}^{-1}{\left(j\omega \right)}^{-n}\;(0\leq n\leq 1)$$

In Equation (8), *Y*_0_ is the CPE-constant, *j* is the imaginary number unit, *ω* is the angular frequency (*ω* = 2*πf*, *f* being the frequency), and *n* is the CPE-power. When *n* = 1, the CPE represents a pure capacitive.

Table 4 shows the EIS results analyzed by ZSimpWin. It can be observed that *R*_c_, *R*_f_ and *R*_t_ decreased with the exposure time. With the aging and corrosion of the coating, chlorine ions and other aggressive ions were more prone to invade the anodic oxide film. The long-term action of aggressive ions led to the local dissolution and destruction of the anodic oxide film, which made the corrosion of the substrate easier, and thus the *R*_t_ decreased. The series inductive resistance, *R*_l_, of the coated samples exposed for 20 years was 6.99 × 10^4^ Ω cm^2^, which indicated that the corrosion products deposited on substrate surface had a certain protective effect on the aluminum alloy substrate.

**Figure 10:**
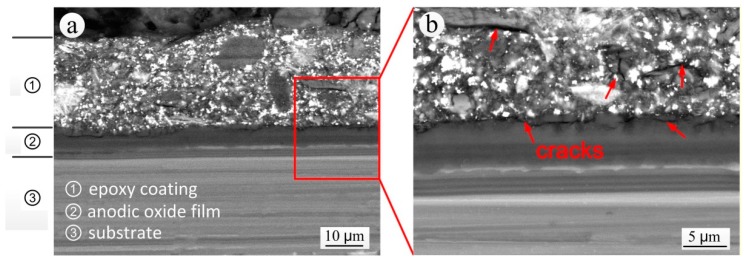
Backscattered electron image of the S-L section of the specimen exposed for 20 years: (**a**) SEM morphology; (**b**) local enlarged SEM morphology.

Figure 10 shows the backscattered electron image of the S-L section of the specimen that was exposed for 20 years. Although there was no visible damage along part of the sectional coating thickness, obvious signs of cracking appeared between the local coating and the anodic oxide film and within the coating, leading to a reduced resistance of the coating to the infiltration of water, chlorine ions, sulfate ions and other aggressive mediums, and thus leaving the substrate very vulnerable to corrosion. This was a good explanation for the apparently contrasting observations made on the coating exposed for 20 years of an apparently intact structure but inductive EIS characteristics. The main causes of cracking were:

(1) Cathodic disbondment: Under the conditions of atmospheric corrosion, the anodic oxide film or aluminum alloy substrate under the coating endures electrochemical corrosion. The oxygen reduction reaction (Equation (9)) occurs on the cathode and the pH in the cathode reign increases significantly. The strong basic environment damages the metal oxide or polymer-coating of the interface, which affects the bonding of the coating to the anodic oxide film, and then causes the cracking [35,36,37].
  (9) $$O_{2}+2H_{2}{O\;+4e}^{-}\to 4{\mathrm{OH}}^{-}$$
(2) Photoaging of coatings: Small molecules such as ketones, alcohols and acids can be washed away by water during photoaging (ultraviolet irradiation), continuously changing the composition of the coating, which therefore contracts and is subject to a decrease in thickness. This causes and embrittlement of the coating [38]. In addition, the loss of the polymer from the coating will effectively increase the volume concentration of the pigments on the surface of coating. Thus, the surface of coating will become relatively brittle while the inner layer of coating is still relatively elastic, which leads to the superficial and inner cracking of the coating.
(3) Temperature alterations: The seasonal and daily alternations of atmospheric temperature cause the expansion and contraction of the coating, and the change of coating tension and internal stress, which leads to the decrease of adhesion, cracking and destruction of coating [39].
(4) Effect of SO_2_ air pollutant: The air pollutant SO_2_ can significantly reduce the adhesion of the coating, resulting in cracking between the coating and anodic oxide film. When SO_2_ and H_2_O are simultaneously present, the destruction of the coating is more obvious [40].

### 3.4. Analysis of the S-L Section of Coating Bubbling Area

**Figure 11:**
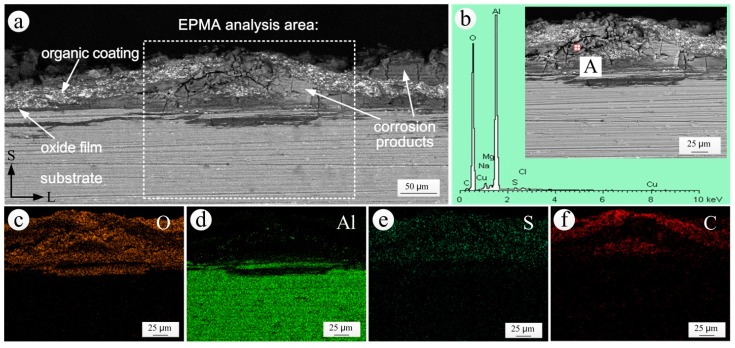
S-L sectional analysis in the coating bubbling area of coated specimen exposed for 20 years: (**a**) SEM morphology; (**b**) EDS spectrum of the corrosion products; (**c**–**f**) the elemental distributions of O, Al, S and C obtained by EPMA.

The S-L sectional SEM morphology and EDS spectrum of the corrosion products and the elemental distribution in the coating bubbling area of coated specimen exposed for 20 years are shown in Figure 11. It can be seen that there was an evident bubbling area in the S-L section of the coated specimen exposed for 20 years (Figure 11a). The anodic oxide film under the bubbling area had been destroyed and the aluminum alloy substrate had also been corroded. The EDS results (Figure 11b) and elemental distributions (Figure 11c,d) revealed that the corrosion products in the interior of the bubbling area of Figure 11a were mainly hydroxides of aluminum. The accumulation of corrosion products between the coating and the substrate and the expansion of their own volume led to the bubbling of the local coating. Sulphur was present throughout the entire coating area, as shown in Figure 11e. The only source of S was the air pollutant SO_2_. The presence of SO_2_ significantly reduced the bonding force between the coating and the substrate, and also reduced the corrosion resistance of the coating [40]. The presence of chlorine in the corrosion products was seen from Figure 11b. The existence of the chlorine ion accelerated the dissolution of the anodic oxide film [41].

In Figure 11f, the epoxy coating, represented by the presence of carbon, was divided into two parts: an upper part and a lower part. This phenomenon may be due to a certain degree of cracking partaking place between the two layers of the inner coating before the corrosion of the aluminum alloy substrate, as shown in Figure 10. After the corrosion of the substrate occurred, the corrosion products moved along the cracks and inside the coating. The coating was then divided into two parts due to the accumulation and expansion of the corrosion products.

### 3.5. Analysis of Exfoliation Corrosion of the Aluminum Alloy Substrate

#### 3.5.1. Exfoliation Corrosion Resistance Analysis of the Aluminum Alloy Substrate

**Figure 12:**
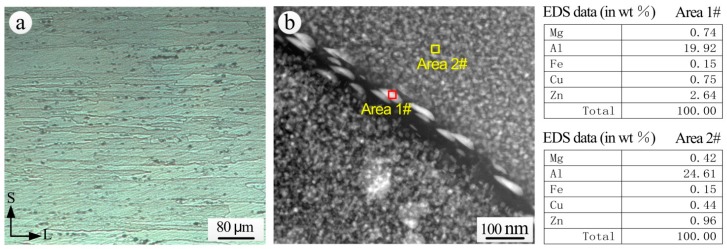
7075-T6 aluminum alloy (**a**) S-L metallographic section; (**b**) grain boundary structure and EDS analysis taken by TEM.

The metallurgical structure of the S-L section and the grain boundary microstructure of 7075-T6 aluminum alloy substrate exposed for 20 years are shown in Figure 12. The grains of extruded 7075-T6 aluminum alloy were flat in the direction of extrusion, and there were a large number of second phase particles (Figure 12a). The second phase particles of 7075 aluminum alloy mainly include η(MgZn_2_), T(AlZnMgCu), S(CuMgAl_2_) and β(Al_7_Cu_2_Fe). The main strengthening phases of 7××× aluminum alloy after T6 aging are the G. P. zone and a small amount of transitional phase $\eta^{\prime }$ (MgZn_2_). A continuous chain of precipitates is distributed at the grain boundary (Figure 12b). From the comparison of EDS analysis results of the intragranular *α* solid solution (Area 2#) and of the precipitates at the grain boundaries (Area 1#), a higher Mg and Zn content is noticed in the grain boundary precipitates, and thus it can be inferred that the grain boundary precipitates consisted mainly of the η phase.

The η phase in the pitting process acts as an anode and will be dissolved preferentially [42]. As the continuous chain of the η phase is distributed at the grain boundary, the anodic η phase at the grain boundary and the precipitate free zone (PFZ) form corrosion interactions and corrosion active channels, resulting in a high susceptibility to intergranular corrosion [43]. Under the action of corrosive mediums, the corrosion propagates along the grain boundaries parallel to the direction of extrusion, and corrosion products accumulate at the grain boundaries. Due to the expansion of corrosion products, wedge forces are generated at the grain boundaries, and eventually the surface of the alloy is exfoliated. Therefore, the 7075-T6 aluminum alloy substrate with 20 years exposure is very sensitive to exfoliation corrosion.

#### 3.5.2. Propagation Characterization Analysis of Exfoliation Corrosion

**Figure 13:**
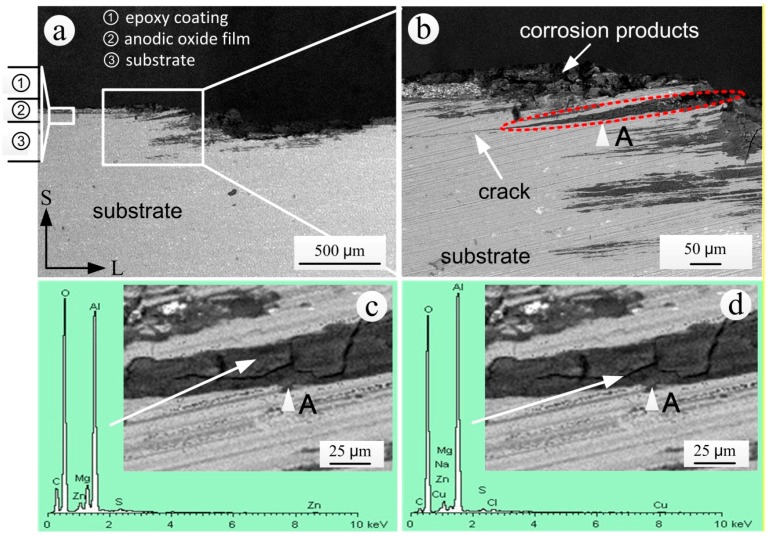
S-L sectional analysis in the junction area between the corroded and un-corroded substrate of the specimen exposed for 20 years: (**a**) SEM morphology; (**b**) local enlarged SEM morphology; (**c**) EDS spectrum in the lump area of the corrosion products; (**d**) EDS spectrum in the cracking area of the corrosion products.

The junction area between the corroded and un-corroded substrate can be used to study the propagation of exfoliation corrosion. Figure 13 shows the S-L sectional SEM morphology and EDS spectrum of corrosion products of the coated specimen that was exposed for 20 years. The substrate and epoxy coating in the left of the exfoliation corrosion area were intact and did not present coating bubbling (Figure 13a). The exfoliation corrosion of the S-L section propagated along the direction of extrusion (L direction). Due to the expansion of intergranular corrosion products, the corrosion products of the A area in Figure 13b showed a wedge shape. Moreover, the structures of the aluminum alloy on the upper side of the corrosion product area noticeably exfoliated outwardly, whereas the exfoliation of structures on the lower side was not obvious. At the same time, there was an obvious crack at the tip of the corrosion products.

Due to the ventilation and daily temperature alternations on the test site, the surface of the specimens underwent dry and wet alternation, which made the products of intergranular corrosion harden (Figure 13c). From the results of EDS analysis in the block area of corrosion product in Figure 13c, it can be found that C and S appeared in the lump area of corrosion products besides the oxygen and the elements contained in the aluminum alloy substrate. Carbon may originate from the organic coating. After local aging and destruction of the organic coating, the polymers entered the corrosion area with the corrosive medium. The existence of S indicated that the air pollutant SO_2_ was indirectly involved in intergranular corrosion through conversion to acid in the moist environment. The annual average of NO_2_ in the Wanning test site was far less than the annual average of SO_2_, as shown in Table 2, correspondingly, N was not found in the corrosion products (Figure 13c,d). Thus, it can be preliminarily inferred that NO_x_ was not involved in the exfoliation corrosion of the aluminum alloy substrate.

The EDS analysis results of the cracking area (Figure 13d) showed the presence of Na and Cl, which were not measured in the lump area (Figure 13c). This result indicated that the cracks in the area of the corrosion products provided the channels for the transmission of corrosion mediums such as NaCl, which created conditions for the further propagation of corrosion.

**Figure 14:**
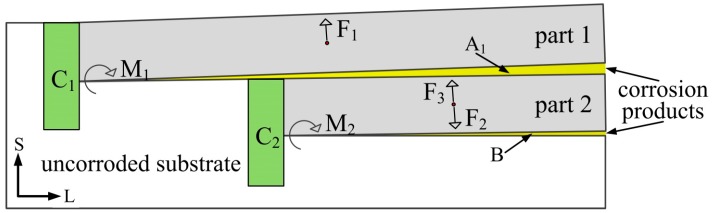
Mechanical model of wedge exfoliation analysis.

The analysis of wedge exfoliation that occurred near the A area in Figure 13b can be simplified into the mechanical model shown in Figure 14. Under the action of the expansion force of the corrosion product in the A_1_ area, structures of aluminum alloy on the upper side (part 1) exfoliated outwardly, and the intergranular corrosion of structures under the lower side (part 2) also occurred along the L direction. Compared to the area of un-corroded substrate, the exfoliation of the structures on both the upper and lower sides of the A_1_ area can be considered as the bending of the cantilever beam under load. For the equilibrium of part 1, the wedging force of corrosion products, F_1_, and the bending moment, M_1_, produced at the cantilever beam base, C_1_, must be in balance. Similarly, for the equilibrium of part 2, the wedging forces of corrosion products, F_2_ and F_3_, and the bending moment, M_1_, produced at the cantilever beam base, C_2_, must be in balance. It was obvious that before the part 1 had been completely exfoliated outwardly, if part 2 appeared the same degree of wedge exfoliation as part 1, it would require a greater wedging force of the corrosion products in the B area. However, due to the fact that the intergranular corrosion in the B area was not deep enough to provide enough wedge force, part 2 does not form the obvious wedge exfoliation as shown in the A_1_ area.

## 4. Conclusions

(1) After exposure for 20 years in the Wanning test sites, the epoxy coating had been partially destroyed, and the exfoliation corrosion had occurred on the extruded 7075-T6 aluminum alloy substrate. The remaining thicknesses of epoxy coatings for macroscopically intact coating areas followed a normal distribution and decreased linearly.
(2) The corrosion resistance of epoxy coatings decreased with the increase of exposure time. After 12 years of exposure, the coating still had a protective effect. After 20 years, although the coating was apparently intact, it had lost its protective characteristics and EIS results showed the occurrence of inductive characteristics. The reason was the formation of cracks between the local coating and the anodic oxide film and of cracks within the coating, which reduced the resistance of the coating to aggressive mediums.
(3) The anodic oxide film under the epoxy coating bubbling area had been destroyed and the aluminum alloy substrate had also been corroded. The corrosion products in the interior of the coating bubbling area were mainly hydroxides of aluminum.
(4) As the continuous chain of η phase was distributed at the grain boundary, the extruded 7075-T6 aluminum alloy was sensitive to exfoliation corrosion. The propagation of exfoliation corrosion in the S-L section was along the direction of extrusion. Cracks between the lumps of corrosion products provided the channels for the transmission of corrosion mediums, which created conditions for the further propagation of corrosion. Moreover, the structures of the aluminum alloy on the upper side of the corrosion product area displayed a more evident exfoliation than the lower side.

## Figures and Tables

**Table 1:** Chemical composition (wt %) of extruded 7075-T6.

Element	Si	Fe	Cu	Mn	Mg	Cr	Zn	Ti	Al
Weight fraction (%)	0.50	0.50	1.68	0.39	2.31	0.18	6.01	0.10	Bal.

**Table 2:** Average environmental data measured in 1997 at Wanning.

Environmental Characteristics	Average (Ranges)
Air temperature(°C)	23.9 (31.4–17.8)
Relative humidity (%)	87.6 (100.0–80.0)
Velocity of wind (m/s)	2.4
SO_2_ (mg/m^3^)	0.0452
NO_2_ (mg/m^3^)	0.0020
Cl^−^ deposition rate (mg/(m^2^ d))	14.5875

**Table 3:** Distribution parameters and correlation coefficients of the distribution models.

Years	Normal			Log-normal			Logistic			Weibull			Gumbel		
	*μ*	*σ*	*r*	*μ*	*σ*	*r*	*μ*	*σ*	*r*	*σ*	*β*	*r*	*μ*	*σ*	*r*
7	39.5	4.0	0.993	1.6	0	0.989	39.5	2.3	0.985	41.2	12.0	0.985	37.6	3.3	−0.955
12	31.9	3.4	0.993	1.5	0	0.991	31.9	1.9	0.990	33.3	11.7	0.983	30.4	2.7	−0.968
20	22.5	2.5	0.986	1.4	0.1	0.972	22.5	1.4	0.985	23.5	11.1	0.995	21.3	2.1	−0.935

**Table 4:** Simulated parameters of EIS results of coated samples exposed for 7, 12, and 20 years.

Samples	*Q*_c_ (F cm^−2^)	*n*_c_	*R*_c_ (Ω cm^2^)	*Q*_f_ (F cm^−2^)	*n*_f_	*R*_f_ (Ω cm^2^)	*C*_dl_ (F cm^−2^)	*R*_t_ (Ω cm^2^)	*R*_l_ (Ω cm^2^)	*L* (H)	Chi-Square Value (*χ*^2^)
7 years	1.01 × 10^−9^	0.90	6.83 × 10^5^	1.24 × 10^−8^	0.74	2.41 × 10^7^	2.57 × 10^−9^	4.61 × 10^7^	2.466 × 10^−3^
12 years	2.64 × 10^−8^	0.75	8.62 × 10^4^	4.61 × 10^−8^	0.81	4.95 × 10^6^	8.62 × 10^−8^	5.38 × 10^6^	1.066 × 10^−3^
20 years	9.51 × 10^−7^	0.59	7.08 × 10^3^	2.10 × 10^−7^	0.89	1.41 × 10^4^	2.05 × 10^−7^	2.49 × 10^5^	6.99 × 10^4^	9.97 × 10^4^	1.134 × 10^−3^
